# Curcumin-Loaded Nanoparticles Based on Amphiphilic Hyaluronan-Conjugate Explored as Targeting Delivery System for Neurodegenerative Disorders

**DOI:** 10.3390/ijms21228846

**Published:** 2020-11-23

**Authors:** Giuseppe Pepe, Enrica Calce, Valentina Verdoliva, Michele Saviano, Vittorio Maglione, Alba Di Pardo, Stefania De Luca

**Affiliations:** 1 IRCCS Neuromed, 86077 Pozzilli, Italy; g.pepe1604@gmail.com (G.P.); vittorio.maglione@neuromed.it (V.M.); 2 Institute of Biostructures and Bioimaging, National Research Council, 80134 Naples, Italy; enrica.calce@gmail.com (E.C.); valentina.verdoliva@gmail.com (V.V.); 3 Institute of Crystallography, National Research Council, 70126 Bari, Italy; michele.saviano@ic.cnr.it

**Keywords:** curcumin delivery, HA-based biomaterial, biodegradable nanoparticles, neuroprotection, Huntington’s disease

## Abstract

Identification of molecules able to promote neuroprotective mechanisms can represent a promising therapeutic approach to neurodegenerative disorders including Huntington’s disease. Curcumin is an antioxidant and neuroprotective agent, even though its efficacy is limited by its poor absorption, rapid metabolism, systemic elimination, and limited blood–brain barrier (BBB) permeability. Herein, we report on novel biodegradable curcumin-containing nanoparticles to favor the compound delivery and potentially enhance its brain bioavailability. The prepared hyaluronan-based materials able to self-assemble in stable spherical nanoparticles, consist of natural fatty acids chemically conjugated to the natural polysaccharide. The aim of this study is to provide a possible effective delivery system for curcumin with the expectation that, after having released the drug at the specific site, the biopolymer can degrade to nontoxic fragments before renal excretion, since all the starting materials are provided by natural resource. Our findings demonstrate that curcumin-encapsulated nanoparticles enter the cells and reduce their susceptibility to apoptosis in an in vitro model of Huntington’s disease.

## 1. Introduction

Over the years, several natural compounds have been explored as adjuvant treatment to the conventional drug therapies for a number of pathological settings. Curcumin (CUR), isolated from *Curcuma long* and commonly used as a traditional remedy for a variety of conditions, exhibits great promise as a therapeutic and effective agent against pathological settings, owing to its diverse therapeutic properties including anti-inflammatory, anti-oxidant, and wound healing properties [1,2].

CUR has been reported also to act as a neuroprotective molecule. It has been tested in several brain disorders including Huntington’s disease (HD) [3,4], a hereditary neurodegenerative condition, caused by the expansion of a polyglutamine tract in huntingtin protein [5]. However, the potential efficacy of CUR is demonstrated to be limited, due to low blood–brain barrier (BBB) permeability, poor absorption and rapid metabolism [6] ultimately resulting in lack of bioavailability. Nanotechnology-based drug delivery vehicles have been proven to improve the bioavailability of CUR and to enhance its efficacy when used as therapy [7,8,9].

Various water-soluble formulations of CUR have been applied to study the ways to increase the retention time of curcumin in the body. Cheng et al. encapsulated CUR within polyethyleneglycol-polylactide (PEG-PLA) di-block polymer micelles in order to obtain an efficient delivery and its incremented bioavailability when treating Alzheimer’s disease (AD) [10]. Curcumin-loaded poly lactic-co-glycolic acid (PLGA) nanoparticles (NPs) have been studied also for their ability to cross the BBB [11] and to induce neural stem cell proliferation and differentiation, either in vitro or in the hippocampus and subventricular zone of adult AD rats [12].

It has been, recently, demonstrated that curcumin encapsulated in solid lipid nanoparticles (C-SLNs) exerts beneficial effects and attenuates neurological defects in a mouse model of HD [13]. In line with that, Del Prado et al. reported on the study performed on micelles of polycaprolactone encapsulating curcumin for neuronal application [14].

In this study, amphiphilic hyaluronic acid (HA)-fatty acid conjugates, previously developed, have been proven to self-assemble in micellar nanoparticle embedding CUR in their inner and to enter cells. In particular, the physiochemical properties, particle size, zeta potential and morphology of the CUR-HA-palmitate NPs have been analyzed. Our results highlight the validity of the CUR-carrier system (CUR-HA-palmitate) by pointing out the reduced susceptibility to apoptosis of HD cells after exposure.

## 2. Results and Discussion

### 2.1. Preparation and Characterization of CUR Loaded HA-Fatty Acid Nanoparticles

**Scheme 1:**
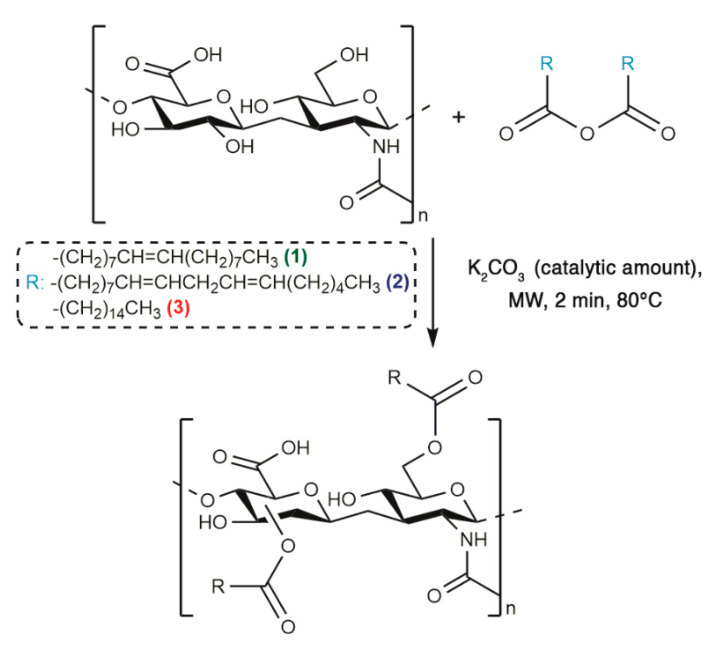
Synthetic strategy of fatty acid-HA-palmitate conjugates.

The optimized synthetic procedure to obtain HA-fatty acid conjugates has been already reported, therefore an MW-assisted esterification strategy in absence of solvent was performed in order to prepare HA-oleate, HA-linoleate and HA-palmitate (Scheme 1) [15,16].

**Figure 1:**
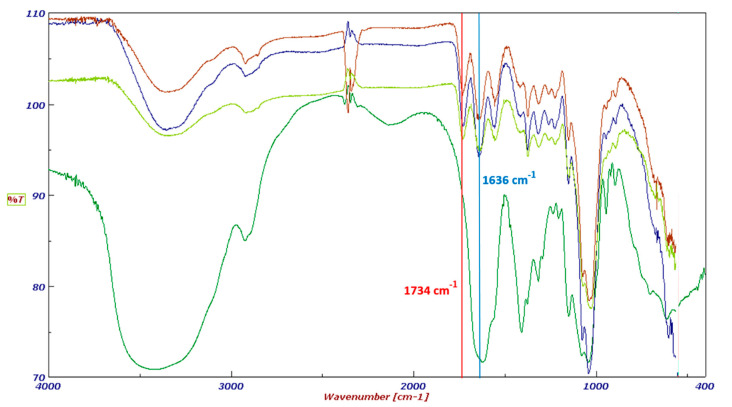
FT-IR spectra of HA (dark green), HA-palmitate (blue), HA-oleate (brown), and HA-linoleate (light green).

The final products were fully characterized by FT-IR spectroscopy. The FT-IR spectra of HA–oleate, HA–linoleate and HA–palmitate are shown in Figure 1; for comparison purpose a spectrum of the native HA is also included. Beside the band (~1636 cm^−1^) of the HA-COO^−^, in the region of the carboxylic group stretching a new band appears (~1734 cm^−1^). It takes into account of the occurred esterification of the fatty acid-HA.

Concerning the tendency of this HA-based material to self-assemble into micelles under physiological conditions, it was already studied by using the pyrene fluorescence method and the critical aggregation concentration was determined in the range of 0.12–0.3 mg mL^−1.^

Starting from the previously investigated HA-conjugates properties as drug delivery nanosystem, the so-called emulsion evaporation method was applied to form HA-based aggregates that simultaneously encapsulate CUR [15,16,17]. It involved the dissolution of the curcumin in an organic solvent (ethanol) and the HA derivatives in an aqueous solution of NaCl 0.9%. A fast shaking movement was applied over-night at room temperature and allowed the breaking of the emulsion and the evaporation of the organic layer. As a result, the aggregates were found dissolved into the aqueous phase and their size distribution was investigated with the dynamic light scattering technique (DLS). The mean diameter of CUR loaded HA-fatty acid nanoparticles ranged from 150 to 500 nm, depending on the fatty acid conjugated to the hyaluronan (Table 1).

As a matter of fact, the particle size of the CUR-HA–oleate and CUR-HA–linoleate resulted bigger than CUR-HA–palmitate, even though it was not expected. In fact, it was previously found that HA conjugated to unsaturated fatty acids were characterized by a smaller particle size, while the opposite tendency is observed for the same nano-particles that encapsulate CUR. It is likely that acid unsaturation modulates the cohesion of the particle in a different manner in case a CUR is embedded into the hydrophobic core.

The zeta potential values of the HA-based nano-particles were negatively charged (from 30 to 28 mV) due to the negative charge of the HA carboxylic groups located in the shell, and these values may prevent the aggregation of particles through electro-static repulsion (Table 1).

**Figure 2:**
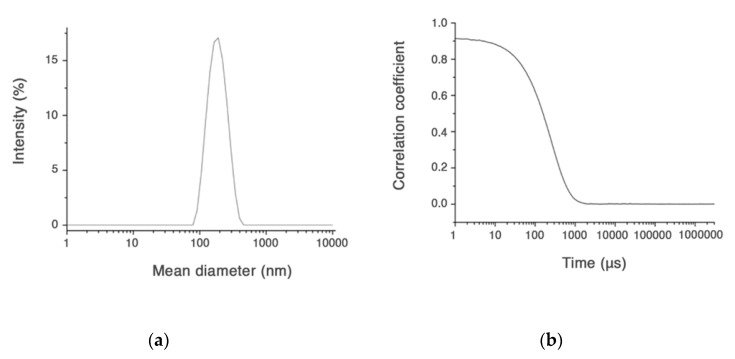
Size distribution (**a**) and intensity correlation functions for CUR-HA-palmitate (**b**).

Only the CUR-HA-palmitate nanoparticles were investigated for their CUR entrapment efficiency, since they had a diameter size <200 nm, which means suitable to cross the neuronal cell membrane (Figure 2).

In major details, the amount of CUR content (final concentration) in HA-palmitate micelles was evaluated using the UV-vis absorption spectroscopy, after disruption of the aggregates in ethanol and evaluation of the freed CUR by reading its absorbance at 420 nm. The concentrations of curcumin considered suitable for the subsequent cellular uptake evaluation experiment were never lower than 1.5 µM (Table 1).

The release kinetic of the curcumin from the Cur-HA-palmitate nanoparticles was evaluated over 72 h at room temperature in phosphate buffer (0.1 M, pH = 7.4). We observed a continuous drug release in the chosen time range, until the saturation was reached after 24 h. It could be estimated that ~70% of the encapsulated curcumin was released after 72 h.

The size and morphology of the prepared nanoparticle were analyzed by transmission electron microscopy technique (TEM), which indicates a spherical shape and an average size range of 150 nm.

**Figure 3:**
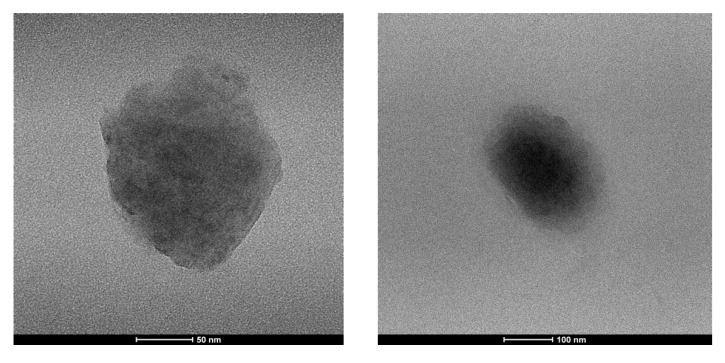
Morphology of CUR-HA-palmitate nanoparticles measured using transmission electron microscopy (TEM).

The slight difference observed for TEM images is not a surprise; nano-system size is somewhat unreliable, since the aggregates flatten and spread on the grid, during the sample preparation, in dry conditions (Figure 3).

### 2.2. CUR-HA-Palmitate Nanoparticles Dysplay Higher Cell Permeability Compared to Pure CUR

Striatal-derived immortalized cell line expressing mutant huntingtin (STHdh^111/111^) represents a widely used HD model for studying molecular aspects of the disease [18,19].

**Figure 4:**
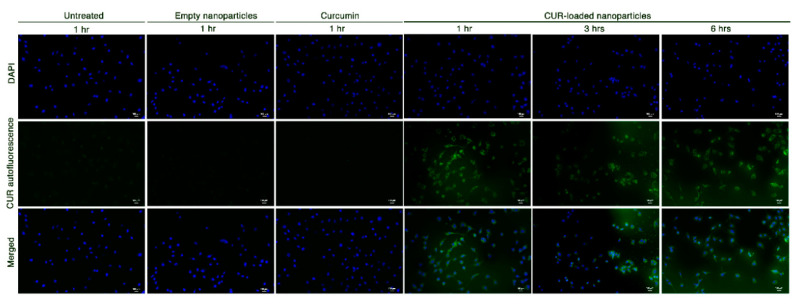
Curcumin green autofluorescence in STHdh^111/111^ HD striatal-derived cells, before and after exposure to empty nanoparticles, 3.8 µM pure curcumin and 3.8 µM CUR-loaded nanoparticles at different time points (scale bar: 100 μm; 20× magnification).

To establish whether the HA-based nanosystems could increase curcumin bioavailability, STHdh^111/111^ cells were cultured in a medium containing either pure curcumin, empty nanoparticles or CUR-HA-palmitate (CUR-loaded) nanoparticles. Subsequently, curcumin-related auto-fluorescence [20] was detected at different time points. As depicted in Figure 4, a green fluorescence signal was detected only in cells treated with CUR-loaded nanoparticles.

### 2.3. Treatment with CUR-HA-Palmitate Nanoparticles Reduces Apoptosis in a Cell Model of HD

A number of pieces of evidence demonstrated that curcumin displays anti-apoptotic action in several cell lines, including neurons [21].

Here, in order to establish whether an increased curcumin cell bioavailability could be associated with any anti-apoptotic effect, striatal-derived STHdh^111/111^ HD cell lines, which usually display high susceptibility to apoptosis [18], were pre-cultured in medium-containing either empty nanoparticles (NANO), pure curcumin (CUR), or CUR-loaded nanoparticles (NANO-CUR), at fixed time points. Cells were successively incubated in apoptotic conditions (serum free medium) [22] and a percentage of dead cells was assessed by Annexin-V assay and cytofluorimetric analysis [18,22].

**Figure 5:**
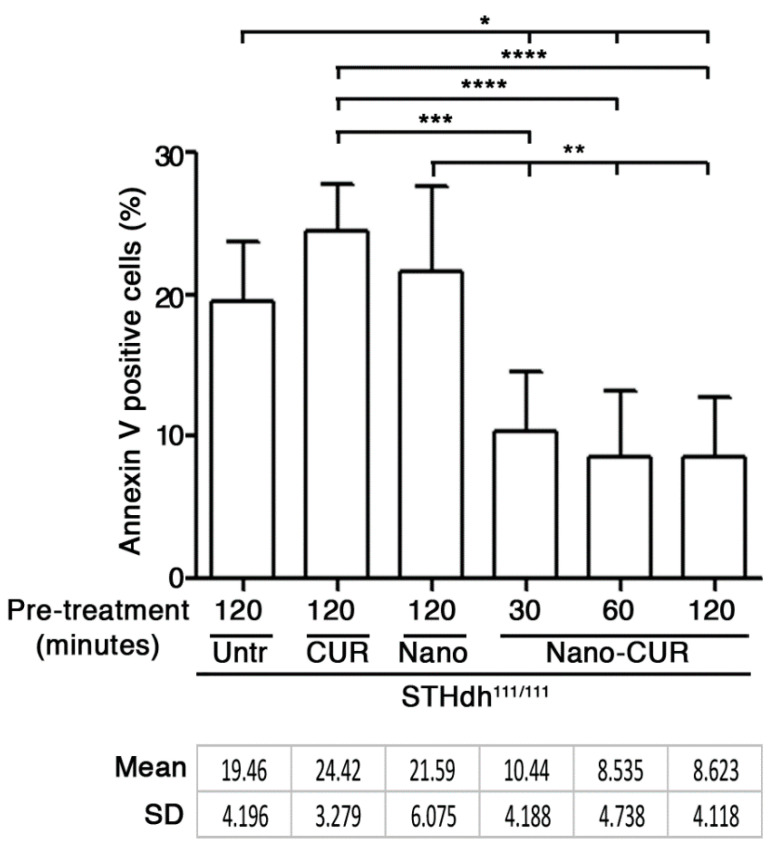
Apoptosis in STHdh^111/111^ HD striatal-derived cells pre-cultured with serum-free medium (Untr) in presence of pure curcumin (CUR), empty nanoparticles (Nano) and CUR-loaded nanoparticles (Nano-CUR) at different time points. Percentage of apoptotic (Annexin-V positive) cells is expressed as mean ± SD of three experiments, each performed in duplicate. * *p* < 0.05; ** *p* < 0.01; *** *p* < 0.001; **** *p* < 0.0001 (One-Way ANOVA with Tukey post-test). Untr: untreated; CUR: curcumin; Nano: empty nanoparticles; Nano-CUR: curcumin-loaded nanoparticles.

As reported in Figure 5, NANO-CUR significantly reduced cell susceptibility to apoptosis. The percentage of apoptotic cells was significantly reduced in all CUR-loaded nanoparticle treatments, independently of the time of the pre-treatment.

No significant effect was detected in cells pre-treated with either pure curcumin or empty nanoparticles.

## 3. Materials and Methods

### 3.1. Compounds

The Hyaluronic acid sodium salt from Streptococcus equi mol wt 15–30 kDa, fatty acids, curcumin and all solvents were purchased from Sigma–Aldrich (Merck, Darmstadt, HE, Germany).

### 3.2. Synthesis and Characterization of HA-Fatty Acids Conjugates

#### 3.2.1. Synthesis of HA-Fatty Acid Conjugates

Fatty acid anhydrides were synthesized as follows. The appropriate fatty acid (10 mmol) was dissolved in dichloromethane (2 mL), the solution was cooled in an ice-water bath and stirred vigorously under argon atmosphere. The dicyclohexylcarbodiimide (5 mmol), previously dissolved in the minimum volume of dichloromethane, was added and the stirring was continued at ice bath temperature for 2 h. The white solid N,N’-dicyclohexylurea was removed by filtration and the solvent was evaporated in vacuum to give the final anhydride. By using an agate mortar, hyaluronan and the appropriate fatty acid anhydride, at the same weight ratio, were manually milled in the presence of catalytic amount of K_2_CO_3_ to obtain several hyaluronan derivatives. The samples, placed in a 0.5–2 mL microwave vial, were irradiated for 2 min at 80 °C in a microwave oven (Initiator, Biotage Sweden AB, Uppsala, Sweden). After cooling at room temperature, the obtained solid was dissolved in water, placed in a 250 mL separatory funnel and extracted with ethyl acetate in order to remove the unreacted fatty acids. Subsequently, the aqueous layer was neutralized by adding 0.5 N HCl solution in water and then dialyzed (membrane cut off 6000–8000) for 1 day in Milli-Q water. The final product was collected after the lyophilization process. Yield: around 40%.

#### 3.2.2. FT-IR Characterization

The modified hyaluronan samples were analyzed by FT-IR spectroscopy. The FT-IR spectra were recorded on a UV-Vis spectrophotometer (Jasco, Easton, MD, USA). Samples were ground into a fine powder using an agate mortar before being compressed into KBr discs. The characteristic peaks of IR transmission spectra were recorded at a resolution of 4 cm^−1^ over a wavenumber region of 400–4000 cm^−1^. The bands relevant for the structural organization of the HA-fatty acid derivatives are as follows. HA-oleate: FT-IR (cm^−1^): 3428 *ν*(O–H), 2923 *ν*(C–H), 1734 *ν*(C=O fatty acid ester), 1636 *ν*_as_(COO^−^), 1413 *ν*_as_(COO^−^), 1078 *ν* and 1040 *ν*(COC)_glycosidic bond ring_. HA-linoleate: FT-IR (cm^−1^): 3419 *ν*(O–H), 2926 *ν*(C–H), 1734 *ν*(C=O fatty acid ester), 1636 *ν*_as_(COO^−^), 1415 *ν*_as_(COO^−^), 1078 *ν* and 1040 *ν*(COC)_glycosidic bond ring_. HA-palmitate: FT-IR (cm^−1^): 3415 *ν*(O–H), 2919 *ν*(C–H), 1734 *ν*(C=O fatty acid ester), 1636 *ν*_as_(COO^−^), 1415 *ν*_as_(COO^−^), 1080 *ν* and 1047 *ν*(COC)_glycosidic bond ring._

### 3.3. Formulation and Characterization of Curcumin-Loaded HA-Fatty Acid Nano-Particles

Curcumin-loaded HA-fatty acid nanoparticles were prepared by solvent evaporation method. In detail, 0.5 mg of curcumin were dissolved in 1 mL of chloroform and added to 3 mg of HA-fatty acid conjugate, previously dissolved in 3 mL of H_2_O 0.9% with NaCl. The two phases were emulsified by stirring over-night at room temperature to allow the organic solvent to evaporate and the nano-particles to form into the aqueous layer. Thereafter, the solution was centrifuged at 13,000 rpm for 10 min and the insoluble unloaded curcumin was removed using a pre-equilibrated Sephadex G50 column (GE Healthcare). Then, the curcumin-loaded HA-fatty acid nano-particles were freeze-dried and stored at 4 °C for further use.

**Figure 6:**
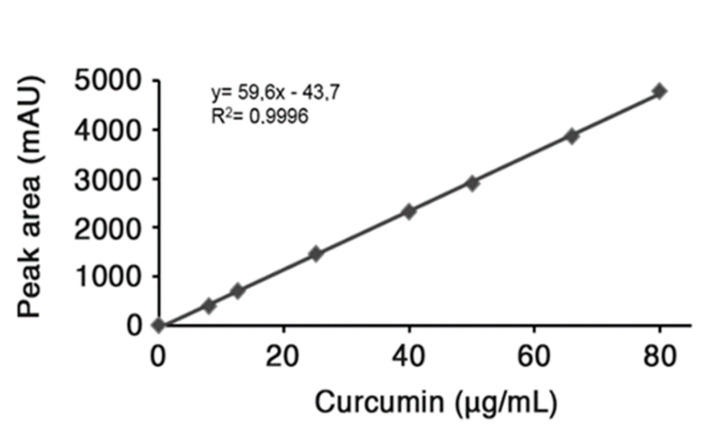
Calibration Curve.

Encapsulation efficiency. The loading amount of curcumin in the nano-particles was evaluated by dissolving predetermined amount of lyophilized drug-loaded nanoparticles in EtOH and sonicating for 20 min; subsequently, the suspension was centrifuged at 10,000 rpm for 10 min. The yellowish supernatant was collected, dried under vacuum and re-suspended in acetonitrile to quantitatively analyze the drug content by HPLC. RP-HPLC analysis was carried out on a HP Agilent Series 1100 apparatus using a reversed phase C-18 column, 4.6 × 250 mm, with a flow rate of 0.5 mL min^−1^. The system solvent used a mobile phase composed of H_2_O 0.1% TFA (A) and CH_3_CN 0.1% TFA (B) with a linear gradient starting from 50% to 90% B in 5 min and detection at 420 nm. The injection volume for drug analysis was 5 µL and the retention time of freed curcumin was 4.6 min. The HPLC peak area of the sample under investigation was evaluated. To determine the curcumin content in HA-fatty acid nano-particles, a standard concentration curve previously determined was used as reference (Figure 6) [23]. In major details, a standard stock solution was prepared using 0.5 mg of free curcumin dissolved in 0.5 mL EtOH; after removal of the organic solvent, the drug was re-suspended in acetonitrile and different concentrations were obtained by successive serial dilutions that were used to perform HPLC analysis and generate a calibration curve (Figure 6). The final value obtained is referred to the curcumin amount (µg) contained in a collected volume of nano-particles, then the value was converted in µM concentration.

#### 3.3.1. In Vitro Curcumin Release Study and Their Dimension Investigation

**Figure 7:**
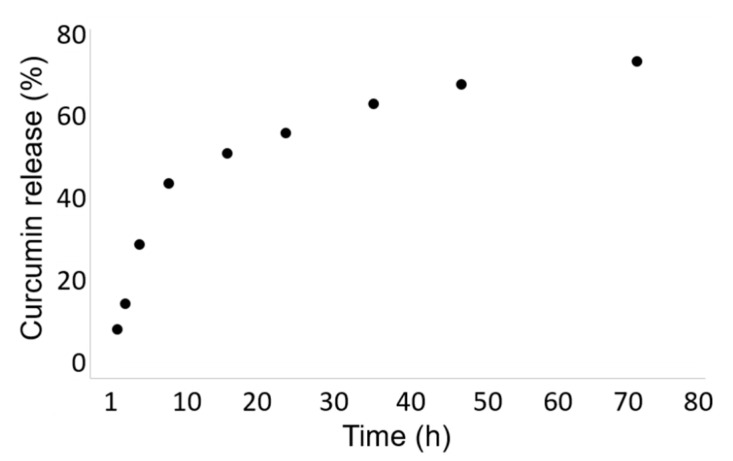
In Vitro Curcumin Release Study.

To investigate the drug release, Cur-HA-palmitate nanoparticles were dissolved in 1 mL of phosphate buffer (0.1 M; pH = 7.4) and placed in a dialysis bag (6 kDa) that soaked in a baker containing the same buffer solution. The dialysis was performed under magnetic stirring for 72 h. At predetermined time intervals, an aliquot (1 mL) of the dissolution medium was withdrawn and its absorbance was measured at 420 nm. The same amount of fresh medium was added to the dialysis container to maintain the sink conditions. As 100% of encapsulated curcumin gave an absorbance of 0.339, it allowed the calculation of the percentage of the released curcumin over 72 h (Figure 7).

The particle size distribution and zeta potential of the curcumin-loaded HA-fatty acid nano-particles were measured at 25 °C by dynamic light scattering (DLS) technique with a Malvern Zetasizer (Nano ZS, Malvern Instruments, Westborough, MA, USA) with NIBS optics.

The scattered light was measured at an angle of 173° and was collected on an autocorrelator. The hydrodynamic diameters (d) of micelles were calculated by using the Stokes–Einstein equation. All data were averaged over three measurements.

#### 3.3.2. Transmission Electron Microscopy

Transmission electron microscopy observation was performed on a high-resolution Transmission Electron Microscope (Tecnai G2 Spirit TWIN, Lausnne, Switzerlan, Europe) operating at an acceleration voltage of 120 kV. The specimen was prepared as follows. One drop of dilute latex was cast on a copper EM grid covered with a thin holey carbon film and dried at room temperature.

#### 3.3.3. Cell Models

Conditionally immortalized mouse striatal knock-in cells expressing endogenous levels of mutant Htt (mHtt) (STHdh^111/111^) were purchased from the Coriell Cell Repositories (Coriell Institute for Medical Research, Camden, NJ, USA) and maintained as previously described [18].

#### 3.3.4. In Vitro Uptake of Curcumin Nanoparticles

Cells were exposed to empty nanosystem and either pure curcumin or curcumin-containing nanoparticles for different times with the concentration of 3.8 µM curcumin in all forms. Cell nuclei were stained with 4′-6 diamidino-2-phenildindole (Vector Laboratories, Burlingame, CA, USA) for 10 min at room temperature. Coverslips were mounted using Prolong Gold antifade reagent (Invitrogen, Life Technologies, Bleiswijk, NN, Netherlands). Images were captured using a fluorescence microscope (Nikon Instruments Europe, Amstelveen, AS, The Netherlands).

#### 3.3.5. Treatment with CUR-Loaded Nanoparticles and Analysis of Apoptosis

Cells were cultured in standard growth medium and pre-treated with either curcumin or CUR-loaded nanoparticles as indicated (see Figure 5) and then maintained under apoptotic conditions (5 h in serum free medium at 39 °C) as previously described [22]. For the analysis of apoptosis, at the end of the treatment, cells were collected and incubated with FITC-conjugated Annexin V (BD, Cat. N. 556419), according to the manufacturer’s instructions. Fluorescence-activated cell sorting (FACS) analysis was performed as previously described [22].

## 4. Conclusions

The beneficial effect of curcumin has been reported in different neurodegenerative conditions including HD [4]. However, its relatively low bioavailability and poor solubility in aqueous solution represent one of the major problems for curcumin delivery. To overcome these problems, different strategies have been applied including the development of nanoparticle-based carriers [24].

In this study, we report on amphiphilic HA-derivatives obtained by chemical conjugation of natural fatty acids to the backbone of the polysaccharide, and the obtained materials were able to self-assemble. In particular a novel formulation of curcumin encapsulated in these micelles has been developed; the CUR-loaded HA-based nanoparticles were characterized for their particle size, zeta-potential, entrapment efficiency, shape and cellular uptake.

Moreover, we demonstrated that CUR-loaded nanoparticles were efficiently up-taken by a well-validated and widely used neuronal-like HD model and any toxic effect was detected.

Conversely, treatment significantly reduced cell susceptibility to apoptosis, further supporting its potential application as neuroprotective agent. In this regard, we have extensively demonstrated that any compound, described to be effective in STHdh cells, was also beneficial in HD animal models and represented a potential therapeutic approach for the disease [22,25,26,27].

We have recently demonstrated that a curcumin-supplemented diet, in pregnant females, was beneficial in HD offspring, by preserving motor performance and reducing huntingtin toxicity [4]. However, no evidence of the therapeutic potential of such approach in adult HD mice was provided.

In light of that, we intended to develop a CUR-loaded HA nanoparticle with good cell bioavailability and with the potential of being administered in symptomatic HD mice.

In spite of the attractive pharmacokinetic advantages [28,29], recent studies have proven the toxic potential of nanodrug delivery systems, since they often exhibit in vitro and in vivo cytotoxicity, oxidative stress, inflammation, and genotoxicity. In this regard, the developed drug carrier systems are based on a natural polysaccharide (HA), characterized by non-toxicity, non-immunogenicity and biodegradability, which is chemically bound to natural fatty acids [30,31]. It allows us to hypothesize a safe circulation for the hyaluronan-based (HA) material, which should guarantee its implementation as a biocompatible nanocarrier for drug delivery in vivo. In this regard, similar HA-based nano-emulsion–containing polyphenols, including curcumin, were proven to be safe and able to reach the mouse brain, through the intranasal route [32].

Although additional studies are needed to investigate the therapeutic potential of our approach in HD, we believe that HA-based nanoparticles may represent a good carrier for the brain delivery of neuroprotective agents. In addition, this approach could provide the opportunity to design novel therapeutic routes for the treatment of HD and, eventually, other brain disorders.

## Figures and Tables

**Table 1:** Characteristic parameters of CUR-HA-fatty acid conjugates.

	HA-Oleate	HA-Linoleate	HA-Palmitate
**Mean diameter (nm)**	430.1 ± 8.2	254.7 ± 12.8	186.7 ± 2.7
**Polydispersity**	0.210 ± 0.08	0.398 ± 0.14	0.236 ± 0.003
***δ* (mV)**	−32.5	−26.2	−30.6
**[CUR]**	2.7 µM	1.2 µM	5.2 µM

## Abbreviations

AD	Alzheimer’s disease
BBB	Blood brain barrier
CUR	Curcumin
C-SLNs	Curcumin-loaded solid lipid nanoparticles
DLS	Dynamic light scattering
EtOH	Ethanol
EM grid	Electron microscopy grids
FACS	Fluorescence-activated cell sorting
FITC	Fluorescein isothiocyanate
FT-IR	Fourier-transform infrared spectroscopy
HA	Hyaluronic acid
HD	Huntington disease
mHtt	Mutant huntingtin
NaCl	Sodium chloride
Nano-CUR	Curcumin-loaded nanoparticles
NPs	Nanoparicles
PEG-PLA	Polyethyleneglycol-polylactide
PLGA	Poly lactic-co-glycolic acid
RP-HPLC	Reversed-phase high performance liquid chromatography
rt	Room temperature
STHdh^111/111^	Mutant Htt-expressing cells
TEM	Transmission electron microscopy
